# Sociodemographic associations with abnormal estimated glomerular filtration rate (eGFR) in a large Canadian city: a cross-sectional observation study

**DOI:** 10.1186/s12882-018-0991-5

**Published:** 2018-08-09

**Authors:** Irene Ma, Maggie Guo, Daniel Muruve, Hallgrimur Benediktsson, Christopher Naugler

**Affiliations:** 1 Department of Pathology and Laboratory Medicine, Cumming School of Medicine, University of Calgary, 9, 3535 Research Rd NW, Calgary, AB T2L 2K8 Canada; 2 Department of Medicine, Cumming School of Medicine, University of Calgary, 9, 3535 Research Rd NW, Calgary, AB T2L 2K8 Canada; 3 Snyder Institute for Chronic Diseases, Cumming School of Medicine, University of Calgary, 9, 3535 Research Rd NW, Calgary, AB T2L 2K8 Canada; 4 Department of Family Medicine, Cumming School of Medicine, University of Calgary, 9, 3535 Research Rd NW, Calgary, AB T2L 2K8 Canada; 5 Department of Community Health Sciences, Cumming School of Medicine, University of Calgary, 9, 3535 Research Rd NW, Calgary, AB T2L 2K8 Canada; 6 Calgary Laboratory Services, Calgary, AB Canada

**Keywords:** Chronic kidney disease, Estimated glomerular filtration rate, Laboratory medicine, Census data, Geospatial mapping

## Abstract

**Background:**

Chronic kidney disease (CKD) is often asymptomatic in its early stages but is indicated and is diagnosed with an estimated glomerular filtration rate (eGFR) < 60 ml/min/1.73m^2^. Certain sociodemographic groups are known to be at risk for CKD, but it is unclear if there are strong associations between these at risk groups with abnormal eGFR test results in Canada. Using only secondary laboratory and Census data, geospatial variation and sociodemographic associations with abnormal eGFR result rate were investigated in Calgary, Alberta.

**Methods:**

Secondary laboratory data from all adult community patients who received an eGFR test result were collected from Calgary Laboratory Service’s Laboratory Information System, which is the sole supplier of laboratory services for the large metropolitan city. Group-level sociodemographic variables were inferred by combining laboratory data with the 2011 Canadian Census data. Poisson regression and relative risk (RR) were used to calculate associations between sociodemographic variables with abnormal eGFR. Geographical distribution of abnormal eGFR result rates were analyzed by geospatial analysis using ArcGIS.

**Results:**

Of the 346,663 adult community patients who received an eGFR test result, 28,091 were abnormal (8.1%; eGFR < 60 ml/min/1.73m^2^). Geospatial analysis revealed distinct geographical variation in abnormal eGFR result rates in Calgary. Women (RR = 1.11, *P* < 0.0001), and the elderly (age ≥ 70 years; *P* < 0.0001) were significantly associated with an increased risk for CKD, while visible minority Chinese (RR = 0.73, *P* = 0.0011), South Asians (RR = 0.67, *P* < 0.0001) and those with a high median household income (RR = 0.88, *P* < 0.0001) had a significantly reduced risk for CKD.

**Conclusions:**

Presented here are significant sociodemographic risk associations, and geospatial clustering of abnormal eGFR result rates in a large metropolitan Canadian city. Using solely publically available secondary laboratory and Census data, the results from this study aligns with known sociodemographic risk factors for CKD, as certain sociodemographic variables were at a higher risk for having an abnormal eGFR test result, while others were protective in this analysis.

## Background

Chronic kidney disease (CKD) represents a significant disease burden, with a prevalence of approximately 12.5% of adult Canadians and 15.2% of Americans affected [1, 2]. Patients living with CKD costs the Canadian health care system over $40 billion CAD per year, with US estimates of over $50 billion USD in Medicare costs [3, 4]. Early detection and management of CKD are critical for reducing comorbidities and mortality rates, as the condition may be reversible. Unfortunately, early identification of CKD is challenging, as patients with early stages of CKD are often asymptomatic, due to the variable and non-specific clinical presentation of the disease [5, 6]. In fact, CKD is often diagnosed serendipitously through other common comorbidities and risk factors, including diabetes mellitus, hypertension, and cardiovascular disease (CVD) [6–8], which often meant their disease has progressed without detection. Individuals with certain sociodemographic factors may also be more vulnerable to developing CKD than others. Evidence show a higher prevalence of CKD in some visible minorities and certain Indigenous populations in Canada and the United States [9–15]. Those with different levels of income, education and employment may also be vulnerable to developing CKD [16–19]. However, it is clear that sociodemographic risk factors for CKD is population- and region-specific, as demonstrated by a study showing that low income, but not level of education, was strongly associated with CKD in the United States, while low education level, but not low income, was strongly associated with CKD in the Netherlands [18].

Despite the challenges of early identification and complexity of CKD risk, CKD is indicated and can be diagnosed when an individual have an abnormal estimated glomerular filtration rate (eGFR) < 60 ml/min/1.73m^2^. According to the most recent guideline released by Kidney Disease: Improving Global Outcomes (KDIGO), adults with eGFR < 60 ml/min/1.73m^2^ is considered to be at a moderately increased risk to very high risk for CKD [6]. When measuring renal function, eGFR is currently the clinical standard worldwide over serum creatinine measurement alone, as age, sex, diet, medication use, muscle mass and body size differences among individuals can significantly affect the interpretation of an abnormal creatinine result [20]. Today, the most widely used equation to estimate GFR is the Chronic Kidney Disease Epidemiology Collaboration (CKD-EPI) eq. (21), which not only accounts for the patient’s serum creatinine values, sex, age and ethnicity, but is also more accurate in diagnosing earlier stages of CKD in patients who have a higher eGFR [21, 22]. As a result, many clinical laboratories use the CKD-EPI eGFR equation to diagnose CKD worldwide [1, 6, 23–25].

The purpose of this study is to evaluate the sociodemographic associations with CKD, a complicated chronic disease, using only one laboratory test result, an abnormal eGFR, in a Canadian healthcare setting. To our knowledge, we are the first to do this using a single laboratory test result for CKD, where we combined secondary laboratory data with publically available Canadian census data for a large metropolitan city of Calgary, Alberta. As CKD risk is population- and region-specific, geographic distribution of abnormal eGFR result rate in the city of Calgary will also be assessed.

## Methods

### Laboratory data sources

Calgary Laboratory Services (CLS) is the sole supplier of laboratory services for Calgary and its surrounding area in Alberta, performing on average 29 million tests annually for a catchment population of approximately 1.4 million individuals. All eGFR test results from adult community patients were obtained from the Laboratory Information System (LIS) from CLS for the 2011 calendar year. 2011 was chosen as at the time the study was conducted, the 2011 Census was the most recent Censu Adult eGFR in Calgary is calculated by using a modified version of the CKD-EPI GFR eq. (21), where the patient’s age, sex and measured blood creatinine value are used in the equation, but not the ethnicity of the patient, as that variable is unattainable from laboratory requisitions. All blood creatinine assays performed in Calgary are calibrated using the isotope-dilution mass spectrometry (IDMS)-traceable method [6]. Thus, any adult community patient who received a creatinine laboratory report from CLS, also received an eGFR report, all of whom were represented in this cross-sectional observation study. Hospitalized patients who received a serum creatinine value were not included in this study. Only the first eGFR test result encounter per community patient was included in the study in order to prevent skewing the data with results from patients who required regular screening or surveillance of renal function using eGFR [26]. Patients with an abnormal eGFR test result (eGFR < 60 ml/min/1.73m^2^) in Calgary were then linked with 2011 Statistics Canada Census data to investigate any sociodemographic associations.

### Census linkage

Group-level sociodemographic variables were inferred by combining laboratory data with Statistics Canada’s 2011 Census data, as described previously [27–30]. Briefly, individual-level sociodemographic variables, including date of birth (DOB), gender and Personal Health Number (PHN) were collected for each patient from the LIS. Each patient’s postal code was then determined using PHNs from the Alberta Health Services Registry. The patient’s postal code allows for linking the patient’s eGFR test results to the 2011 Canadian Census Dissemination Units (DU) in the city of Calgary. In Statistics Canada’s census data, DUs exist as polygons that represent the smallest geographical units containing 400–700 individuals within each dissemination area throughout Canada [31]. Patient confidentiality was not an issue in this study because potentially identifying information, such as PHN, were removed for the analysis of this study. Patient data with missing PHN were excluded from the study as the PHN is required to link to the 2011 Canadian Census. Group-level sociodemographic variables were then inferred from the DU within the city of Calgary. Group-level sociodemographic variables for this study were chosen based on plausible associations with abnormal eGFR or CKD [25, 32], which included: recent immigrant status (immigration within the last 5 years), Indigenous status (First Nations, Métis; 2 largest Indigenous populations in Calgary [33]), visible minorities (including “Black”, “South Asian”, “Chinese”; the 3 largest visible minority populations in Calgary [33]), employment rate (percentage of individuals employed), education level (individuals with at least some university education), and median household income (MHI). Each variable (age, gender, ethnicity, socioeconomic status (SES)), was individually controlled for by holding all other variables constant as a reference. Therefore, each variable studied in this model has all been adjusted for.

### Statistical analysis

A Poisson regression (SAS v.9 software) was performed to identify any statistically significant associations between the sociodemographic factors (independent variable) and the proportion of abnormal eGFR test results, or known as the abnormal eGFR result rate (dependent variable) per DU. Abnormal eGFR result rate was calculated by dividing the number of abnormal eGFR test results (as defined by eGFR < 60 ml/min/1.73m^2^) by the total number of the first eGFR test encounter for the calendar year within each DU. The sociodemographic associations were represented by the risk ratio (RR), where a value over 1.0 suggested the independent variable in question had a higher risk of abnormal eGFR result rate when compared to its reference state. For individual-level variables, all age groups < 69 years of age and the female gender were compared to a reference state of age ≥ 70 year and the male gender, respectively. Age was broken down into 7 groups (see Table 1). Each group-level sociodemographic variable was compared to its respective reference state. For example, all Indigenous populations from the 2011 Census groups not included in the study were used as a reference for First Nations and Metis groups in this study. Therefore MHI was represented in units of $100,000 CAD. *P* < 0.05 was deemed statistically significant (SAS v. 9 software).

**Table 1:** Abnormal eGFR result rates in Calgary, Alberta in 2011

	eGFR Test Result (*n*)	Abnormal eGFR Test Result (*n*)	Abnormal eGFR Result Rate (%)
Total	346,663	28,091	8.1%
Female	195,844	16,412	8.4%
Age < 20 years	2,305	6	0.3%
Age 20–29 years	21,616	111	0.5%
Age 30–39 years	31,565	288	0.9%
Age 40–49 years	38,423	835	2.2%
Age 50–59 years	41,529	1,962	4.7%
Age 60–69 years	28,613	3,076	10.8%
Age ≥ 70 years	31,793	10,134	31.9%
Male	150,819	11,679	7.7%
Age < 20 years	1,522	4	0.3%
Age 20–29 years	10,724	88	0.8%
Age 30–39 years	20,224	254	1.3%
Age 40–49 years	30,614	727	2.4%
Age 50–59 years	37,052	1,580	4.3%
Age 60–69 years	26,841	2,444	9.1%
Age ≥ 70 years	23,842	6,582	27.6%

### Geospatial analysis

To determine if geospatial distribution variations exists for abnormal eGFR result rate in Calgary, this was plotted onto a map of Calgary for each DU polygons, and was graphically illustrated using ArcGIS v.10.3, as described previously [28, 30, 34]. The Getis-Ord Gi* statistic [35] produced z-scores and *p*-values for each of the 1500 DU polygons within Calgary, which determined statistical significance of hot (high abnormal eGFR result rate) or cold (low abnormal eGFR result rate) spots in 90, 95 and 99% confidence levels within DUs.

### Ethics statement

Ethics approval for this study was received from the University of Calgary Conjoint Health Research Ethics Board (Ethics ID REB 13–0862).

## Results

In 2011, 346,664 adult community patients received an eGFR test result, but one patient was excluded from the study as their DOB was missing. Of the 346,663 adult patients who received their first eGFR test encounter for the 2011 calendar year (56.5% female, 43.5% male), 28,091 patients had an abnormal result (eGFR < 60 ml/min/1.73m^2^). The abnormal eGFR result rate in Calgary in 2011 was 8.1% (8.4% female, 7.7% male). See Table 1 for further details.

In order to investigate sociodemographic associations with abnormal eGFR test results, sociodemographic risk factors known to be associated with CKD were studied. All individual level and group level sociodemographic variables analyzed were adjusted for, thus making any significant associations an independent risk contribution. Poisson regression revealed that women and the elderly (age ≥ 70 years) were significantly associated with an increased risk for eGFR < 60 ml/min/1.73m^2^, suggesting increased risk for CKD. Meanwhile, Chinese, South Asians, and those with a high MHI had a significantly reduced risk for an abnormal eGFR result. For every $100,000 increase in MHI, risk for abnormal eGFR test results significantly decreased by 12%. Refer to Table 2 for further details.

**Table 2:** Association of sociodemographic variables with the abnormal eGFR result rate (eGFR < 60 ml/min/1.73m^2^)

Sociodemographic Variables	Risk Ratio (RR)	95% CI	Chi-Square	*P*-value
Female	1.11	1.09–1.14	88.46	<.0001
Male^a^	1.00	Reference		
Age < 20 years	0.01	0.004–0.01	225.78	<.0001
Age 20–29 years	0.02	0.018–0.02	2766.70	<.0001
Age 30–39 years	0.04	0.03–0.04	5802.60	<.0001
Age 40–49 years	0.08	0.07–0.08	8742.50	<.0001
Age 50–59 years	0.15	0.15–0.16	10088.00	<.0001
Age 60–69 years	0.34	0.33–0.35	4902.60	<.0001
Age ≥ 70 years^b^	1.00	Reference		
Recent immigrant (within 5 years)	1.03	0.82–1.29	0.05	0.8213
Aboriginal – First Nations	1.09	0.78–1.51	0.24	0.6221
Aboriginal – Métis	0.90	0.48–1.69	0.10	0.7463
Visible minority Chinese	0.73	0.60–0.88	10.72	0.0011
Visible minority South Asian	0.67	0.59–0.76	35.41	<.0001
Visible minority Black	1.15	0.85–1.55	0.84	0.3608
Median household income ($100,000 CAD)	0.88	0.85–0.92	38.94	<.0001
Employment rate	0.89	0.77–1.03	2.39	0.1222
At least some university education	0.88	0.77–1.01	3.36	0.0670

^a^ Males were used as a reference group for females

^b^ Age group ≥70 years of age were used as a reference group for all other age groups

**Fig. 1:**
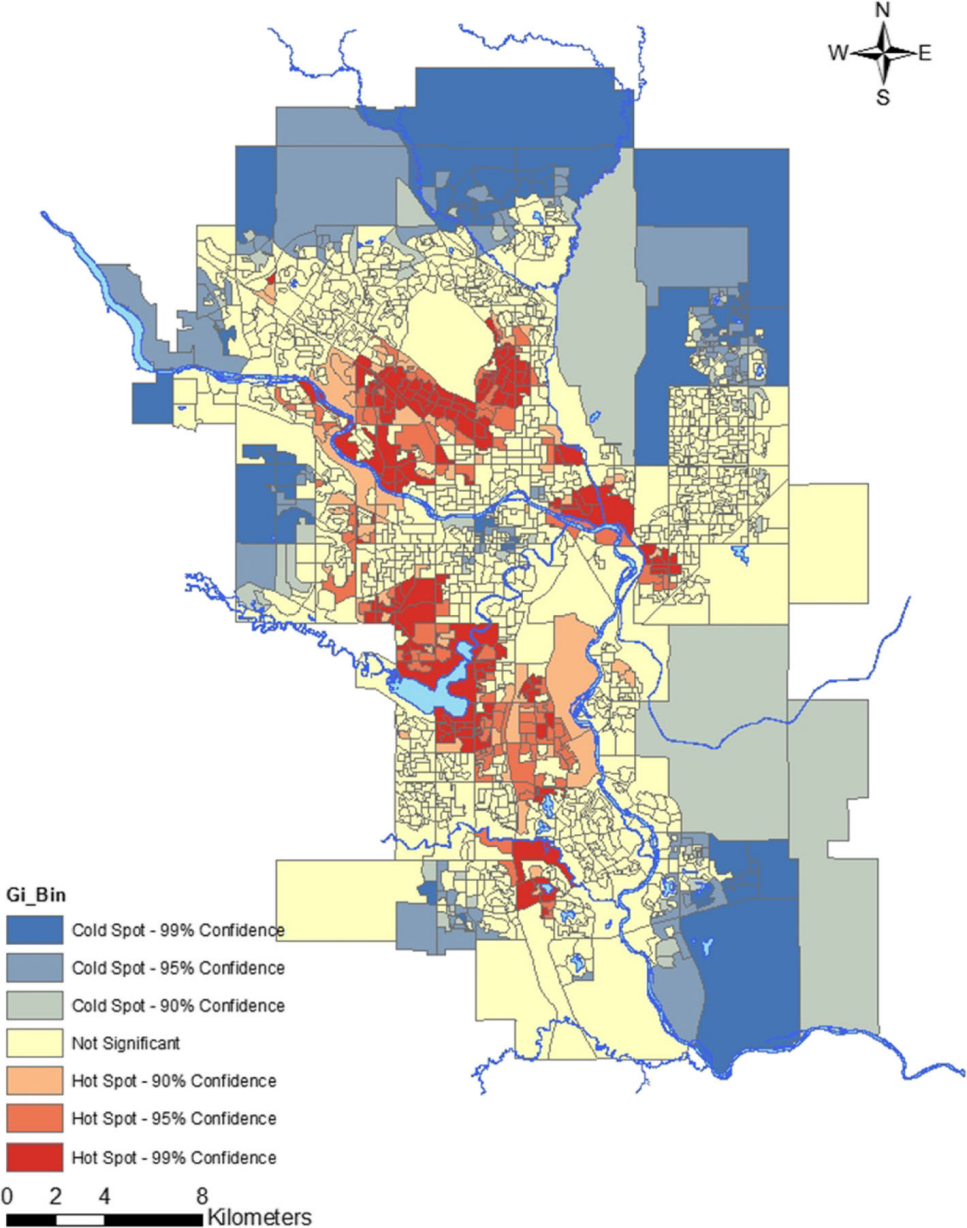
Geospatial variation in abnormal eGFR testing rate in Calgary, 2011. Statistically significant hot spots (red; high abnormal eGFR result rate) or cold spots (blue; low abnormal eGFR result rate) in the 90–99% confidence levels were mapped using the Hot Spot Analysis tool. Statistically significant hot spots clustered in the northwest and southwest quadrant of Calgary (specifically south of Nose Hill Park and surrounding the Glenmore Reservoir), in addition to the communities along the Bow River, east of downtown Calgary. Statistically significant cold spots appeared in the inner city of Calgary, as well in some communities in the periphery of the city

In order to investigate geographic distribution of abnormal eGFR result rate, geospatial analysis was conducted (Fig. 1), and revealed statistically significant regional variations of abnormal eGFR result rates in Calgary, with statistically significant hot spots (high abnormal eGFR result rates) in the northwest, and southwest quadrants of the city of Calgary; as well as along the Bow River, just east of downtown Calgary. Significantly cold spots (low abnormal eGFR result rates) was observed in the inner city of Calgary on the southwest end of downtown, and the outer edges of the city.

## Discussion

In this article, we present geospatial distribution, and sociodemographic risk associations with an abnormal eGFR result rate, suggesting the presence of chronic kidney disease. The methodology of combining secondary laboratory data with Statistics Canada’s Census data (27–29) revealed statistically significant differences in geospatial distribution of abnormal eGFR result rates within Calgary, Alberta. Statistically significant risk associations for abnormal eGFR result rates were also observed for specific sociodemographic groups in the city. To our knowledge, we are the first to validate known sociodemographic risk factors for CKD using a single laboratory test result – an abnormal eGFR.

The geospatial analysis conducted in Calgary demonstrated clear geographic differences in abnormal eGFR result rates, as supported by the regional-specificity of CKD prevalence associated with different sociodemographic factors [9–11, 13, 16–18, 36, 37]. It has been previously demonstrated that certain sociodemographic groups cluster in different parts of Calgary: those with a higher MHI and a higher level of education tend to live in the inner city of Calgary near downtown, while those with lower income, lower level of education, self-reported visible minorities and First Nations groups cluster in the northeast quadrant along the Bow River [28, 38]. This may explain why we observed significant cold (low proportion of eGFR < 60 ml/min/1.73m^2^ result rates) and hot (high proportion of abnormal eGFR result rates) spots in these neighbourhoods, respectively. Another speculation as to why there are significant cold or hot spots in the remaining neighbourhoods, is that perhaps it is due younger families that tend to live in the newer neighbourhoods in the periphery of the city, while those living in the northwest and southwest quadrants in the hot spot areas may overlap with an older demographic. It is unlikely that these hot spots of abnormal eGFR result rates represent greater access to healthcare, as previous studies have shown that in Calgary, Alberta, those living in the neighbourhoods within the periphery of the city travel significantly shorter distances than those in the periphery of the city to phlebotomy sites [39], suggesting easier access to receive laboratory testing for those living inside the city limits. It is also unlikely the hot spots identified in the geomapping analysis represent higher population density, as Statistics Canada standardizes each DU into 400–700 individuals per polygon unit throughout the country [31]. However, as we could not quantitatively assess the sociodemographic associations with abnormal eGFR result rates from the geomapping model alone, we performed a separate analysis (Table 2) to investigate this.

Consistent with other findings, as reviewed by Cobo, et al. [40], there is a statistically significant higher risk of women and the elderly with abnormal eGFR (< 60 ml/min/1.73m^2^) in Calgary, with age having the strongest risk association. This is likely due to age-related changes, and the difference in muscle mass between genders, as both variables impact serum creatinine values, which influences eGFR measurement [41]. Also consistent with other studies [17–19, 42], our analysis showed having a higher income reduces the risk of having an abnormal eGFR result, suggesting a decreased risk for CKD. Specifically, for every increase of $100,000 in MHI, the risk for developing CKD decreases by 12% in our analysis. Although not significant, being employed and having higher levels of education also show a decreased risk in abnormal eGFR result rate in this study. As expected, those who have a higher income, who are employed and who have a higher level of education in Canada tend to be healthier, as these sociodemographic factors influence health-related behaviours [43].

Interestingly, those who are Chinese or South Asian (East Indian, Pakistani, Sri Lankan [44]) are at a statistically significant lower risk of abnormal eGFR result rates compared to other ethnicities, suggesting a lower risk for CKD compared to other visible minorities reported in Census Canada. This result is difficult to interpret due to the fact that some guidelines state that certain ethnicities, including “Asians”, are a risk factor for CKD progression. However, rarely is the “Asian” population clearly defined in other international guidelines [24, 25], whereas in this study, we observed strong sociodemographic associations with specific self-identified visible minorities. Perhaps there was a limitation to our ecological analysis as studies have recommended using a different eGFR equation to include an Asian coefficient to account for the difference in muscle mass between different ethnicities [45, 46], which is not accounted for in the widely used CKD-EPI eq. (21). Finally, there may be possible co-segregation with other unmeasured socioeconomic or clinical variables that also decreased the risk of CKD for these visible minorities in Calgary.

Although there was an increased risk for abnormal eGFR result rates in First Nations and visible minority Black populations in Calgary, the analysis did not show statistical significance. This is interesting as many studies find those who are Indigenous Canadian or from a Black visible minority are more susceptible for CKD, including end stage renal disease (ESRD) [10, 12, 17, 36, 47]. The discrepancies may be due to the fact that Indigenous Canadians who live in an urban centre, such as Calgary, have a higher socioeconomic status [48] and have a decreased prevalence of CKD that is associated with easier access to healthcare [10] when compared to Indigenous Canadians living in more rural areas of the province. It is also important to note that although there was a higher prevalence of Indigenous First Nations with ESRD in Alberta, this was not the case for earlier stages of CKD when compared to non-Indigenous First Nations [9]. Additionally, statistical significance may not have been reached due to the relatively small sample size of First Nations and Black visible minority groups living in Calgary, at < 3% of Calgarian population [9, 33, 48].

By investigating sociodemographic risk associations with an abnormal eGFR result rate using publically available secondary laboratory and Census data, we observed similar known sociodemographic risk associations with CKD. As health is related to socioeconomic status (SES) [43], it is unlikely there is a wide-spread selection bias of eGFR testing for adult community patients in Calgary, as we only observed one of the three SES to have a significantly reduced risk of an abnormal eGFR result, suggesting a reduced risk for CKD.

### Limitations

One limitation to our study is that we did not analyze other common risk factors or comorbidities associated with CKD, such as diabetes, CVD, or hypertension [6, 8]. However, most patients with CKD are not identified or diagnosed until at a later stage of CKD when symptoms are present, or through accidental findings from comorbidities associated with CKD [5, 6, 8]. Thus, eGFR measurement alone allows primary care providers to identify sociodemographic groups at risk for CKD at an earlier stage, and can potentially reverse CKD progression through prompt interventions. Another limitation in the study is that the LIS in CLS does not include the African American, or any ethnicity related coefficient in the CKD-EPI eq. (21), as information regarding ethnicity of the patient cannot be collected from laboratory requisitions. It may be of interest for researchers for Canadian jurisdictions to modify the CKD-EPI equation to account for our diverse population, as others have done [46, 49]. Moreover, as our model included the first eGFR test encounter for the calendar year, our analysis may have included patients who had their eGFR test repeated for confirmation of CKD diagnosis, or have an advanced stage of CKD, also known as ESRD. Therefore, although we reported an abnormal eGFR result rate of 8.1% in Calgary in 2011, we were not able to investigate true CKD prevalence in this analysis. In future, it may be of interest to separate out the different categories of CKD, rather than including an abnormal eGFR flag of 0–60 ml/min/1.73m^2^. Despite these limitations, our model of combining secondary laboratory data with Census data resulted in sociodemographic risk associations with abnormal eGFR result rates, which was validated by, and aligned with, known sociodemographic risk association with CKD.

## Conclusions

In this cross-sectional observation study, there were statistically significant region-specific variations in abnormal eGFR result rates in a large Canadian city. Those who are female, or over the age of 70 were at a significantly higher risk for CKD, while those of visible minority Chinese or South Asian groups, or have a high median household income, were at a significantly lower risk for CKD in this analysis. Results from this study may be useful for policy makers to allow primary care providers to target at risk sociodemographic groups and neighbourhoods identified in this study through decreased eGFR for early identification of CKD progression. This study validated the methodology of using publically available census data and secondary laboratory data to investigate factors associated with CKD risk, which could be applied to investigate sociodemographic associations with other chronic disease conditions using only secondary laboratory data. It may be of interest to analyze and compare results from this study with other jurisdictions across Canada using the same model, as the results shown here may be specific to this particular jurisdiction.

## Abbreviations

CKD: Chronic kidney disease
CKD-EPI: Chronic kidney disease epidemiology collaboration
CLS: Calgary laboratory services
CVD: Cardiovascular disease
DOB: Date of birth
DU: Dissemination unit
eGFR: Estimated glomerular filtration rate
ESRD: End stage renal disease
IDMS: Isotope-dilution mass spectrometry
LIS: Laboratory information system
MHI: Median household income
PHN: Provincial health number
RR: Relative risk
SES: Socioeconomic status

## References

[1] Arora P, Vasa P, Brenner D, Iglar K, McFarlane P, Morrison H (2013). Prevalence estimates of chronic kidney disease in Canada: results of a nationally representative survey. CMAJ.

[2] Centers for Disease Control and Prevention. Chronic Kidney Disease Surveillance System - United States [Internet]. Vol. 2017. Available from: https://nccd.cdc.gov/CKD/. Accessed 29 Sept 2017.

[3] Honeycutt AA, Segel JE, Zhuo X, Hoerger TJ, Imai K, Williams D (2013). Medical costs of CKD in the Medicare population. J Am Soc Nephrol.

[4] Manns B, McKenzie SQ, Flora A, Gignac PM, Geller LI, Canadians Seeking Solutions and Innovations to Overcome Chronic Kidney Disease (Can-SOLVE CKD) Network (2017). The financial impact of advanced kidney disease on Canada Pension Plan and private disability insurance costs. Can J Kidney Heal Dis.

[5] Campbell D, Weir MR (2015). Defining, Treating, and Understanding Chronic Kidney Disease--A Complex Disorder. J Clin Hypertens.

[6] Kidney Disease (2013). Improving Global Outcomes (KDIGO) CKD Work Group. KDIGO 2012 Clinical Practice Guideline for the Evaluation and Management of Chronic Kidney Disease. Kidney Int.

[7] Levey AS, Coresh J, Balk E, Kausz AT, Levin A, Steffes MW (2003). National Kidney Foundation practice guidelines for chronic kidney disease: evaluation, classification, and stratification. Ann Intern Med.

[8] Levin A, Hemmelgarn B, Culleton B, Tobe S, McFarlane P, Ruzicka M (2008). Guidelines for the management of chronic kidney disease. CMAJ.

[9] Gao S, Manns BJ, Culleton BF, Tonelli M, Quan H, Crowshoe L (2007). Prevalence of chronic kidney disease and survival among aboriginal people. J Am Soc Nephrol.

[10] Komenda P, Lavallee B, Ferguson TW, Tangri N, Chartrand C, McLeod L (2016). The Prevalence of CKD in Rural Canadian Indigenous Peoples: Results From the First Nations Community Based Screening to Improve Kidney Health and Prevent Dialysis (FINISHED) Screen, Triage, and Treat Program. Am J Kidney Dis.

[11] Vassalotti JA, Li S, McCullough PA, Bakris GL (2010). Kidney early evaluation program: a community-based screening approach to address disparities in chronic kidney disease. Semin Nephrol.

[12] Choi AI, Rodriguez RA, Bacchetti P, Bertenthal D, Hernandez GT, O’Hare AM (2009). White/black racial differences in risk of end-stage renal disease and death. Am J Med.

[13] Lora CM, Daviglus ML, Kusek JW, Porter A, Ricardo AC, Go AS (2009). Chronic kidney disease in United States Hispanics: a growing public health problem. Ethn Dis.

[14] Reddan DN, Szczech LA, Klassen PS, Owen WF (2000). Racial inequity in America’s ESRD program. Semin Dial.

[15] Yeates K (2010). Health disparities in renal disease in Canada. Semin Nephrol.

[16] Bello AK, Peters J, Rigby J, Rahman AA, El Nahas M (2008). Socioeconomic status and chronic kidney disease at presentation to a renal service in the United Kingdom. Clin J Am Soc Nephrol.

[17] Hall YN, Choi AI, Chertow GM, Bindman AB (2010). Chronic kidney disease in the urban poor. Clin J Am Soc Nephrol.

[18] Vart P, Gansevoort RT, Coresh J, Reijneveld SA, Bultmann U (2013). Socioeconomic measures and CKD in the United States and The Netherlands. Clin J Am Soc Nephrol.

[19] Krop JS, Coresh J, Chambless LE, Shahar E, Watson RL, Szklo M (1999). A community-based study of explanatory factors for the excess risk for early renal function decline in blacks vs whites with diabetes: the Atherosclerosis Risk in Communities study. Arch Intern Med.

[20] Stevens LA, Coresh J, Greene T, Levey AS (2006). Assessing kidney function--measured and estimated glomerular filtration rate. N Engl J Med.

[21] Levey AS, Stevens LA, Schmid CH, Zhang YL, Castro AF, Feldman HI (2009). A new equation to estimate glomerular filtration rate. Ann Intern Med.

[22] Levey AS, Bosch JP, Lewis JB, Greene T, Rogers N, Roth D (1999). A more accurate method to estimate glomerular filtration rate from serum creatinine: a new prediction equation. Modification of Diet in Renal Disease Study Group. Ann Intern Med.

[23] Primary Care Education Advisory Committee for KHA (PEAK). Chronic kidney disease (CKD) management in clinical practice: guideline and clinicla tips to help identify, manage and refer patients with CKD in your practice [Internet], vol. 2017. 3rd ed: Kidney Health Australia; 2014. Available from: https://kidney.org.au/cms_uploads/docs/ckdm-in-gp-handbook.pdf. Accessed 29 Sept 2017.

[24] National Insititute for Health and Care Excellence (NICE). Chronic kidney disease in adults: assessment and management (Clinical Guideline 182) [Internet], vol. 2017: NICE Guideline; 2014. Available from: https://www.nice.org.uk/guidance/cg182/resources/chronic-kidney-disease-in-adults-assessment-and-management-pdf-35109809343205. Accessed 29 Sept 2017.

[25] Inker LA, Astor BC, Fox CH, Isakova T, Lash JP, Peralta CA (2014). KDOQI US commentary on the 2012 KDIGO clinical practice guideline for the evaluation and management of CKD. Am J Kidney Dis.

[26] McFarlane P, Gilbert RE, MacCallum L, Senior P (2013). Canadian Diabetes Association 2013 clinical practice guidelines for the prevention and management of diabetes in Canada. Chronic kidney disease in diabetes. Can J Diabetes.

[27] de Koning L, Henne D, Woods P, Hemmelgarn BR, Naugler C (2014). Sociodemographic correlates of 25-hydroxyvitamin D test utilization in Calgary, Alberta. BMC Heal Serv Res.

[28] Gorday W, Sadrzadeh H, de Koning L, Naugler C. Association of sociodemographic factors and prostate-specific antigen (PSA) testing. Clin Biochem. 2014;47(16–17):164–9. 10.1016/j.clinbiochem.2014.08.006 25130956

[29] Crouse A, Sadrzadeh SM, de Koning L, Naugler C (2015). Sociodemographic correlates of fecal immunotesting for colorectal cancer screening. Clin Biochem.

[30] Griener TP, Naugler C, Chan WW, Church DL. Sociodemographic correlates of urine culture test utilization in Calgary, Alberta. BMC Urol. 2018;18(1):2. 10.1186/s12894-018-0315-x PMC5759274 29310636

[31] Dissemination area (DA) - Census Dictionary [Internet]. 2015. [cited 2018 Jul 11]. Available from: http://www12.statcan.gc.ca/census-recensement/2011/ref/dict/geo021-eng.cfm.

[32] Kazancioglu R (2013). Risk factors for chronic kidney disease: an update. Kidney Int Suppl.

[33] Statistics Canada. 2013. Calgary, CMA, Alberta (Code 825) (table). National Household Survey (NHS) Profile. 2011 National Household Survey. Statistics Canada Catalogue no. 99-004-XWE. Ottawa. Released September 11, 2013. http://www12.statcan.gc.ca/nhs-enm/2011/dp-pd/prof/index.cfm?Lang=E. Accessed 7 Aug 2018.

[34] Barber J, Guo M, Nguyen LT, Thomas R, Turin TC, Vaska M, et al. Sociodemographic Correlates of Clinical Laboratory Test Expenditures in a Major Canadian City. Am J Clin Pathol. 2017;148(1):91–6. 10.1093/AJCP/AQX052 28605433

[35] Mitchell A (2005). The ESRI Guide to GIS analysis, Volume 2: spatial measurements and statistics.

[36] Tarver-Carr ME, Powe NR, Eberhardt MS, LaVeist TA, Kington RS, Coresh J (2002). Excess risk of chronic kidney disease among African-American versus white subjects in the United States: a population-based study of potential explanatory factors. J Am Soc Nephrol.

[37] Thomas MC, Weekes AJ, Broadley OJ, Cooper ME, Mathew TH (2006). The burden of chronic kidney disease in Australian patients with type 2 diabetes (the NEFRON study). Med J Aust.

[38] Naugler C, Zhang J, Henne D, Woods P, Hemmelgarn BR (2013). Association of vitamin D status with socio-demographic factors in Calgary, Alberta: an ecological study using Census Canada data. BMC Public Health.

[39] Baskin L, Abdullah A, Guo M, Naugler C. Use of geospatial mapping to determine suitable locations for patient service centers for phlebotomy services. Am J Clin Pathol. 2015;144(5):727–30. 10.1309/AJCP4J1XKDVJIUGS 26486736

[40] Cobo G, Hecking M, Port FK, Exner I, Lindholm B, Stenvinkel P (2016). Sex and gender differences in chronic kidney disease: progression to end-stage renal disease and haemodialysis. Clin Sci.

[41] James GD, Sealey JE, Alderman M, Ljungman S, Mueller FB, Pecker MS (1988). A longitudinal study of urinary creatinine and creatinine clearance in normal subjects. Race, sex, and age differences. Am J Hypertens.

[42] McClellan WM, Newsome BB, McClure LA, Howard G, Volkova N, Audhya P (2010). Poverty and racial disparities in kidney disease: the REGARDS study. Am J Nephrol.

[43] Mikkonen J, Raphael D (2010). Social determinants of health: the Canadian facts.

[44] Statistics Canada. 2011. 2011 National Household Survey: Immigration and Ethnocultural Diversity. Statistics Canada Catalogue no. 99-004-XWE. Ottawa. Released May 8, 2013. https://www150.statcan.gc.ca/n1/en/catalogue/99-010-X2011028. Accessed 7 Aug 2018.

[45] Ma YC, Zuo L, Chen JH, Luo Q, Yu XQ, Li Y (2006). Modified glomerular filtration rate estimating equation for Chinese patients with chronic kidney disease. J Am Soc Nephrol.

[46] Rule AD, Teo BW (2009). GFR estimation in Japan and China: what accounts for the difference?. Am J Kidney Dis.

[47] Babayev R, Whaley-Connell A, Kshirsagar A, Klemmer P, Navaneethan S, Chen SC (2013). Association of race and body mass index with ESRD and mortality in CKD stages 3–4: results from the Kidney Early Evaluation Program (KEEP). Am J Kidney Dis.

[48] Wei T, Hansen J, Office of Statistics and Information - Demography. National Household Survey - Aboriginal People in Alberta: A Regional Perspective: Alberta Gov Treas Board Financ; 2016. p. 1–12. Available from: http://www.finance.alberta.ca/aboutalberta/osi/census/2011/2011-national-household-survey-release1-Aboriginal-Peoples.pdf. Accessed 9 Mar 2018.

[49] Stevens LA, Claybon MA, Schmid CH, Chen J, Horio M, Imai E (2011). Evaluation of the Chronic Kidney Disease Epidemiology Collaboration equation for estimating the glomerular filtration rate in multiple ethnicities. Kidney Int.

